# Difficulty beyond intubation

**DOI:** 10.4103/0019-5049.68381

**Published:** 2010

**Authors:** Sekar Michael

**Affiliations:** Department of Anaesthesia, Vinayaka Missions Medical College, Karaikal, Pondicherry, India

Sir,

A six-year-old girl was posted for a posterior pharyngeal wall tumour excision. The patient was submitted for preanaesthetic review with chest X-ray as well as X-ray of head and neck. There was no gross abnormality in either and no history suggestive of sleep apnoea.[1] However, it was decided to induce with inhalational anaesthesia anticipating difficulty in intubation.[2] Intubation was easy only with 4.0 uncuffed instead of 6.0 cuffed endotracheal tube that is appropriate for her age group.

As the surgery was underway, there was difficulty in ventilating the child and it was found that the tube had come off as the tumour was being resected, giving a big passage larger than the existing endotracheal tube size (4.0 uncuffed). Since the field was bloody and the saturation kept falling, a cricothyroid puncture was made to oxygenate and later intubation with a 6.0 cuffed tube after clearing the bloody field.

Changing the tube with a different size, as the tumour was resected, was not spontaneous, though it was anticipated and planned for a difficult airway management. This may be a good lesson for the beginners who involve in oropharyngeal wall tumour surgeries.
